# Neoantigen Cancer Vaccines: Generation, Optimization, and Therapeutic Targeting Strategies

**DOI:** 10.3390/vaccines10020196

**Published:** 2022-01-26

**Authors:** Carson R. Reynolds, Son Tran, Mohit Jain, Aru Narendran

**Affiliations:** Departments of Oncology and Pediatrics, Cumming School of Medicine, University of Calgary, Calgary, AB T2N 1N4, Canada; carson.reynolds@ucalgary.ca (C.R.R.); son.tran@ucalgary.ca (S.T.); mohit.jain@ucalgary.ca (M.J.)

**Keywords:** neoantigen, neoepitope, cancer immunotherapy, cancer vaccination, dendritic cell vaccine, personalized vaccine, immunogenic target, adjuvant therapy

## Abstract

Alternatives to conventional cancer treatments are highly sought after for high-risk malignancies that have a poor response to established treatment modalities. With research advancing rapidly in the past decade, neoantigen-based immunotherapeutic approaches represent an effective and highly tolerable therapeutic option. Neoantigens are tumor-specific antigens that are not expressed in normal cells and possess significant immunogenic potential. Several recent studies have described the conceptual framework and methodologies to generate neoantigen-based vaccines as well as the formulation of appropriate clinical trials to advance this approach for patient care. This review aims to describe some of the key studies in the recent literature in this rapidly evolving field and summarize the current advances in neoantigen identification and selection, vaccine generation and delivery, and the optimization of neoantigen-based therapeutic strategies, including the early data from pivotal clinical studies.

## 1. Introduction

Immunotherapeutic approaches to treat cancer have been rapidly advancing in recent years, both in terms of understanding their mechanism of action as well as their clinical relevance and utility. In particular, neoantigen vaccine-based therapeutics development has seen a significant advancement due to its potential to provide an effective and highly tolerable treatment approach to patients with relapsed or refractory malignancies. The objective of this review is to identify and summarize novel experimental findings as they relate to neoantigens, as well as to describe recent advancements to bring neoantigen-based vaccines to the clinic.

## 2. Methods

To conduct this review, a free-word literature search was done using the PubMed database and the data described from 1 January 2015 to 7 July 2021 were analyzed. All data involving neoantigen-based studies in significant detail, clinical or experimental, were included. Results that only discussed neoantigens as a minor component were not included. 

## 3. Neoantigens

Neoantigens are antigens that are expressed in cancer cells, representing the mutations or alterations that have occurred during the transformation process. These molecules are truly unique to the cancer tissue and carry the potential to be recognized by the immune system leading to specific anti-tumor activity by the host [1]. Neoantigens are the aberrantly expressed peptides that result from mutations in the genome that are translated to produce viable peptides. They serve as mechanisms for the immune system to discern self from tumor tissue and have been recognized as a novel target for cancer immunotherapies [2]. Additionally, the mutagenic nature inherent in many cancers indicated the distinct possibility that certain peptides will be altered and can be exploited as ways of selecting the malignant cell population. Historically, however, it has been difficult to identify molecules that are exclusively expressed in tumor cells and not present in normal cells. This has led to the targeting of tumor-associated antigens (TAAs); antigens that are expressed in abnormally large quantities in tumors but still expressed to some degree in normal cells [3]. The efficacy of targeting TAAs is limited by the fact that the body generally will have an immune tolerance to their expression, or that the TAAs have to be expressed in “expendable” tissue, such as reproductive tissue, and therefore treatments targeting TAAs have a characteristic toxicity [3]. In certain cancers, tumor-specific antigens (TSAs) are expressed (i.e., antigens that are expressed exclusively in cancer cells) [4]. However, TSAs are rare and are seldom expressed in all patients. Increasing knowledge in genomics, bioinformatics, and related fields has resulted in the discovery and effective targeting of neoantigens [5].

Historically, there have been several limiting factors in neoantigen research, particularly with respect to the methods to identify effective neoantigens and, as a result, very few processes to identify viable therapeutic neoantigen targets existed previously [5,6]. As genomic analysis began to be used in clinical settings, the prospect of developing focused methodologies that target the specific mutations in cancer cells became more feasible.

With the advent of next-generation sequencing (NGS), entire genomes can be sequenced rapidly allowing large throughput data to be analyzed with high efficiency. In combination with advanced bioinformatic analytical tools, neoantigens can be predicted and entire personalized vaccine reagents can become available for administration in an acceptable time-period [6]. Challenges remain for research in neoantigen vaccines, and they follow behind the more available immunotherapeutic approaches such as checkpoint inhibitors and chimeric antigen receptor engineered (CAR-T) cell therapies.

## 4. Immunotherapies for Cancer

Presently, several therapeutic options exist in the field on cancer immunotherapy. While some forms of immunotherapy are more established clinically, all forms of immunotherapeutic research and development have been accelerated in recent years and have shown promise for future clinical applications [7,8]. These include approaches using adaptive cell transfer and immune checkpoint inhibitors.

### 4.1. Adoptive Cell Transfer Therapy

Adoptive cell transfer (ACT) therapy is one of the more advanced fields of cancer immunotherapy (reviewed in [8,9,10]). This treatment approach focuses on the generation of cancer cytotoxic T cells based on autologous T lymphocytes produced ex vivo [9]. However, this form of therapy has been expanded to more accurately target tumor cells based on engineered T cell receptors that are encoded by genetically modified retroviruses, generating T cells with known cytotoxic properties based on their T cell receptors (TCRs) [10].

More recently, the focus has shifted to cover CAR-T cell therapy [11]. The genetically engineered receptors are chimeric as they contain both antigen-binding and T cell activating functions [12]. There is a hinge component that allows them flexibility in recognition of their specified ligand [10], and they can recognize a wide variety of ligands based on the encoded single-chain variable fragment. Ligands can be used to identify cancer cells such as TSAs or TAAs, but they can also be tumor-associated molecules used to increase T cell activity and overcome a level of immunological tolerance [10]. Because of the nature of the single-chain variable fragment being the engineered receptor’s binding domain, CAR-T cells can often be engineered to activate in a major histocompatibility complex (MHC)-independent manner [9], which is another mechanism of overcoming immunological tolerance. Because TCRs in CAR-T cell therapy are synthetic, it allows the specific targeting of certain antigens, meaning that neoantigen research may be translatable to this form of therapy in the future.

### 4.2. Immune Checkpoint Inhibitors

During the genesis and evolution of the cancer cells, there are several points at which the T cells, under normal circumstances, would initiate cytotoxic activity to eliminate the tumor. Cancer cells, however, have various mechanisms of evading the activation of T cells, and immune checkpoint inhibition, is used to target this evasion [13]. Aberrantly expressed checkpoint proteins inhibit the activation of anti-tumor T cells. Checkpoint inhibition targets this pathway, allowing the T cell activation process to progress. Common clinically used checkpoint inhibitor treatments include the targeting of programmed cell death protein 1 (PD-1), its ligand (PD-L1), and CTLA4 [13]. PD-1 inhibitors target the PD-1 protein found on T cells which binds to PD-L1 which is regularly expressed by many types of normal cells that do not need to be lysed [13]. This has led to the toxicity of checkpoint inhibitor therapies and remains a challenge in its clinical usage [14]. Checkpoint inhibitors are commonly used in a number of clinical trials concurrently with neoantigen vaccines [15,16,17,18,19,20,21].

## 5. Types of Cancer Vaccination

### 5.1. Dendritic Cell Vaccines

Dendritic cell (DC) vaccines use autologous DCs to generate an immune response by exposing the DCs ex vivo to neoantigens (reviewed in [22]). There are multiple ways in which DCs can be made to recognize and present antigen to other immune cells. Tumor antigen-presenting DCs are commonly generated by pulsed exposure of antigens to DCs. For example, tumor lysate containing relevant antigens can be used to pulse DCs, generating a wide array of tumor antigen carrying DCs that may include neoantigens [23]. Another way is to identify and synthesize neoantigen peptides and pulse DCs with the synthetic peptides [24]. Similar to using tumor lysate, a hybrid of tumor cell and DC can also be made [25,26], similarly generating a wide array of tumor-based antigens that can activate T cells.

### 5.2. mRNA Vaccines

Much like other vaccines in clinical use, mRNA vaccines provide an individual’s cells with the genetic material to make the antigens internally (reviewed in [27]). In the context of cancer vaccines, antigen-presenting cells (APCs) such as DCs are used. Once the antigenic peptides have been made, the DCs effectively present, in the context of MHC, to relevant immune cells for subsequent activation.

### 5.3. Peptide Vaccines

Peptide vaccines are similar to mRNA vaccines in that they provide the desired antigens in vivo in order to have DCs present them (reviewed in [28]). In the context of neoantigen vaccines, peptides are identified through the use of tumor cell sequencing and bioinformatic tools followed by synthesis. They are then given to patients, often with agents to promote the uptake of the peptides by DCs and recruit immune cells to the area of administration for interaction and subsequent immune activation.

## 6. Neoantigen Identification and Selection

Neoantigen vaccination is a promising concept but requires a significant amount of logistical support and infrastructure to generate clinically usable material. Often, the generation of personalized genomic data required for this process can be a limiting factor. Several databases and libraries of neoantigens have been constructed and are currently available for this purpose [24,29,30,31]. Furthermore, as more studies are recognizing shared mutations across separate tumors [29,32,33,34,35,36,37], databases and libraries will become increasingly important, providing highly usable information to identify and validate neoantigen sequences in the future.

Several recent reports have described the process for the identification of tumor type specific neoantigens, with some studies focusing on the feasibility of generating patient specific neoantigens. For example, a study on metastatic melanoma found a total of 26 neoepitope targets from a combined 21,066 possible mutants [38]. Out of the 26 targets, only one neoepitope tested to be reactive in studies using the patient cells. The low specificity of neoantigen identification is recognized within the field and has directed investigations into novel identification methods meant to improve the viability of therapeutic neoantigen targeting [39,40,41,42,43].

More recent efforts in neoantigen identification through genomic analysis consist of improving the accuracy and reliability of neoantigen predicting tools. The tools usually have a strong focus on the viability of neoantigens based on human leukocyte antigen (HLA) haplotyping, binding affinity, and approaches accounting for non-synonymous mutations [44,45]. Of these tools, however, machine learning algorithms and neural networks appear to be the most promising [46,47,48,49]. They can include various prediction factors and use novel information to enhance existing procedures to score candidate targets. These include an algorithm to minimize false positives [46], increasing positive predictive value [48], and to improve binding predictions [47].

The utilization of data from whole-exome sequencing (WES), mRNA microarrays, and publicly available prediction algorithms have also been used to enhance neoantigen prediction capabilities [50]. It has also been demonstrated that it is possible to identify neoantigens through the analysis of a public mutation library as opposed to performing analyses on each individual patient [29], demonstrating the usefulness of public mutation libraries.

### 6.1. Variations on Traditional Identification and Selection Methods

To complement existing NGS-based methods for neoantigen identification, various studies have investigated additional quantifiable measures to improve neo-epitope prediction scoring. Novel methods described include peptidome analysis [32,51,52], differentiation from self-antigens [53,54], similarity to viral or bacterial antigens [54,55], RNA expression [51,56], and spatial variation in antigen recognition [57,58]. Additionally, novel advances in the knowledge of neoantigen mechanisms has also indicated that methodologies may have to be even more varied in the future to account for factors other than what is traditionally considered for the peptides [59,60].

Neoantigens may not only be expressed due to mutations in DNA, but also due to factors affecting RNA expression. Accordingly, one recent study looked at the possibility of personalized neoantigen prediction based on alternative RNA splicing, and found that neoantigen peptides predicted using the method had improved immunogenicity compared to neoantigens found through conventional means [56]. Another study introduced the Prioritizing of RNA Editing-based Peptides (PREP) [52], which draws upon current knowledge of RNA editing to determine possible neoantigens and subsequently applies predicted HLA binding affinity to further rank possible neo-epitopes. It has been proven to correctly identify certain neoantigens in ovarian cancer. Further supporting the targeting of errors in RNA production, it has been found that those frameshift peptides produced by errors in RNA as opposed to DNA can have improved immunogenicity in certain situations and can even be shared [32]. In some cases, the search for viable neoantigens can even be focused to genetic regions encoding specific proteins such as survivin [33]. These techniques streamline many aspects of candidate peptide identification, although their reliability and practicality in a clinical setting must be further investigated.

One of the most recent advancements in the identification of neoantigens is the recognition of how antigen-HLA association may affect the physical configurations of the involved structures. Due to the lack of reliability of current neo-epitope predicting algorithms, improvements must be made to encompass all factors relating more fully to neoantigen-HLA binding. For example, spatial arrangements of neo-epitopes [33,57], as well as the location of mutated residues in the neoepitope, can be used to predict neoantigens [57,61]. This can be modelled and would contribute to the existing HLA binding affinity prediction algorithms. In fact, one study found that mutated anchor residues on epitopes are conservative, and including this knowledge in a prediction algorithm contributed to increased accuracy in neoantigen identification [61]. Additionally, an atom-based method has been used to assess the propensity of the HLA-peptide complex to bind with TCR based on conformational changes that happen after the HLA-peptide association [58].

### 6.2. Mass Spectrometry

Mass spectrometry (MS) is a widely used method of identifying, and sometimes validating [52], neoantigen targets (reviewed in [62]). For example, MS may be used in cases where the genetic viability of any given neoantigen does not completely account for how the predicted antigen will bind to HLA, or how the antigen-HLA complex will physically present itself to T cells. This approach is often coupled with some form of genomic analysis and most studies use MS to some degree [62]. Mass spectrometry is wide in scope and has a large throughput allowing it to be one of the ideal tools for identifying the most neoantigen targets possible [62]. The technique has been combined with liquid chromatography as demonstrated by Abelin et al. [63] to become even more accurate and outline a more thorough understanding of antigen presentation than what is currently known. The contribution of MS to the understanding of antigen presentation in the context of neoantigens has been further supported by the discovery that the technique can identify HLA binding motifs–the physical manner by which HLA molecules bind to antigens [64].

It is likely that selected combinations of the neoantigen identification methods currently available will be used to obtain the most efficient neoantigen prediction pathways in the future. As current knowledge of machine-learning and bioinformatics advances, neoantigen identification should be modified to account for the increasingly complex array of factors which determine neoantigen viability. An ideal system would aim to build on present knowledge, like MARIA (major histocompatibility complex analysis with recurrent integrated architecture), a multimodal recurrent neural network that predicts the probability of antigen-presentation based on HLA alleles using a variety of factors [47]. In addition to traditional methods to calculate binding affinities, MARIA helps to predict HLA ligands based on MS, antigen expression levels, and protease cleavage signals.

## 7. Delivery of Vaccines

The field of vaccination-based immunotherapy is constantly evolving and novel findings that improve on the efficacy and uptake are continuously reported. One important aspect of the technology is that of vaccine delivery systems. Recent reports on the topic of improving delivery of neoantigen vaccines include the inclusion of immune priming molecules that augment the bodily response, novel technologies that can improve upon the stability of vaccine-related molecules such as liposomes or nanoparticles, and the inclusion of technologies such as photothermal molecules that affect the release of tumor antigens [65].

### 7.1. Immune Priming

Like the various methods of delivery, the inclusion of molecules that supplement immune action and recognition of antigens is vital to vaccine-based therapies. Blass and Ott [66] have recently reviewed the current approaches for immune priming in vaccine therapies. One example of a recent improvement in this approach includes the use of cholesterol-modified antimicrobial peptide DP7-C in combination with a dendritic cell vaccine [67]. The addition of DP7-C was found to improve several aspects of antigen presentation in a mouse model such as antigen uptake from monocyte derived DCs, antigen presentation, and presence of mature monocyte-derived DCs [67]. This study also combined the molecule with a liposome for an mRNA vaccine and found significantly higher DC uptake than the control [68], further supporting the use of DP7-C as an immunoadjuvant. Another example is the conjugation of synthetic neoantigen peptides to ligands for toll-like receptors (TLR), acting as agonists to the receptors and further stimulating immune response [69]. It has also been demonstrated that the fusion of MIP3-α to TAAs improved DC uptake of a DNA vaccine in mice [70].

Another approach to overcoming immune tolerance is to prime immune cells as the vaccine is being delivered. For example, indoleamine 2,3-dioxygenase (IDO) has been included with the rationale of inhibiting cells that promote regulatory cycles as opposed to cell death cycles [71]. It was found that producing an IDO-silenced mRNA vaccine induced T cell responses to target antigens and diversified the immune response, with the patient’s immune system identifying several melanoma-associated antigens. As indicated by Feng et al. [72], diverse immune responses are important and can be crucial prognostic indicators. Another study reported the successful overcoming of immune tolerance to self-antigens found in tumors by co-stimulating CD40 with monoclonal antibodies and TLR agonists [73], echoing the findings that, in murine models, CD40, along with a soluble antigen and a TLR3 agonist, can be an effective immune priming treatment for DC vaccines [74]. Their inclusion resulted in 60% of the T cell population being specific for a cancer epitope and complete clearance of the tumors. Similarly, an experimental nanovaccine that also used TLR agonists as adjuvants found that the vaccine induced complete regression in 70% of tumors [75].

Viral and bacterial vectors have also been used recently as a method of enhancing immune responses to delivered antigens and for delivering genetic information for immune priming. For example, an oncolytic virus containing plasmids for GM-CSF, and shRNA for TGF-β, elicited a strong enough immune response to overcome the immune tolerance of MelanA and demonstrated delayed melanoma growth [76]. It has also been shown that self-replicating RNA generated from adenovirus mechanisms combined with synthetic replicon technology can increase the longevity of RNA in vivo and results in increased uptake [77]. In one study, with the goal of repurposing existing immune cells to target tumor cells, human cytomegalovirus (HCMV) was used as a platform [78]. The virus normally elicits an unusual number of virus specific CD8+ T cells and thus has been theorized as a possible vehicle for retargeting a large number of existing CD8+ cells [78]. Although the vaccine generated a previously uncharacterized block of MHC presentation, it was found that fusion of a CD8+ T cell-stimulating epitope to the virus effectively reactivated existing T cells [78]. Interestingly, it has been found that HCMV antigens elicit cross-reactivity to certain melanoma antigens [54]. Similarly, adenovirus plasmids coding for both tumor-specific antigens and tetanus toxoid can utilize the pre-existing immunity that is commonly present because of routine vaccinations [79]. Furthermore, this approach has demonstrated synergy with PD-1 inhibitors. It has also been shown that oncolytic virus plasmids have the capability of coding for PD-L1 inhibitors themselves, allowing for neoantigen recognition by T cells [80]. Additional experiments have tested the efficacy of linking neoantigen peptides to diphtheria toxin and HBV platforms with varying results [81,82].

The use of mesoporous silica microrods (MSR), which have previously been utilized in vaccine technology, has also been investigated as a method of improving antigen uptake and presentation [83]. The investigators combined MSR with polyethyleneimine (PEI), a molecule that can stimulate pro-inflammatory cytokine production and immune responses when complexed with glycoproteins. This approach was noted to affect tumor growth while also demonstrating synergistic properties with the checkpoint inhibitor, anti-CTLA4 [83]. Furthermore, it has been shown that the simultaneous delivery of a tumor-associated antigen with TLR agonists in a microparticle can enhance immune responses to the tumor-associated antigen in mice [69]. The microparticles protect encased antigenic peptides from degradation, and the delivery of two toll-like receptor agonists co-stimulate DCs to present the antigens and induce stronger T cell responses to the targeted cancer. It has also been shown that DC uptake of neoantigens can be improved by presenting the antigenic peptides as trimers via solid phase peptide synthesis [84]. In ex vivo studies, trimerized antigens outperformed long peptides with equivalent amounts of epitopes in inducing an immune response. To improve upon existing trimer synthesis strategies, a novel small molecule was introduced called Antigen MAtriX (AMAX) which improved the yield, purity, and solubility when compared to conventional trimer synthesis strategies [84].

### 7.2. Nanovaccines

While nanovaccines already exist, their translational viability is now being tested in neoantigen vaccines, commonly merging several vaccine technologies [69,75,80,85,86,87]. A key barrier to overcome in vaccinology is how to effectively include complex adjuvant molecules and simultaneously deliver peptides with various surface charge properties. Lipid nanoparticle vaccine delivery studies have shown that nanoparticle vaccines can overcome that barrier [88]. The vaccines can also utilize charged, or pH-dependent, particles to increase DC uptake [89]. The nanoparticles serve as backbones for the conjugation of various molecules that would otherwise remain unconjugated. It can also increase uptake by giving the particles properties that molecules such as DNA, RNA, or peptides cannot possess in a traditional vaccine form. Nanoparticles have also been demonstrated to increase DC uptake by allowing peptides to effectively travel to lymph nodes, and subsequently target DCs for uptake [86]. Nanoparticles also allow for the delivery of genetic information, as demonstrated by a complex synthesis of DNA and RNA, and subsequent intertwining that has been shown to allow DNA, RNA, and peptides to be in the same nanoparticles [90].

In addition to enhancing immune priming, nanoparticles also have the capability of co-delivering several immune priming molecules simultaneously. For example, they can be used to deliver potent immune receptor agonists for TLRs 7/8 and 9, which have demonstrated the potentiality to induce complete regression in murine models [75], as well as molecules that stimulate other immune paths, such as STING, by using stimulatory agonists [80,87]. Both strategies demonstrated the capacity to enhance neoantigen-specific T cell responses and even the potential to induce tumor regression [75,87]; however, one study only achieved regression with combinatorial checkpoint inhibitor therapy [80]. Another study using nanoparticles found that carrying CpG adjuvants significantly increased priming of CD8+ T cells, but could not affect the frequency of tumor-infiltrating lymphocytes (TILs) [89]. Upon local administration of STING agonist (separately from the nanoparticle), TILs were increased, and the vaccine could induce complete regression in murine models of colon cancer and melanoma [89]. The local administration of STING agonists post-vaccination possibly hints at the mechanism by which TILs are induced. It has also been shown that the immune system can be primed indirectly by stimulating processes that recruit immune cells such as reactive oxygen species (ROS) production. One example is Manganese-doped silica nanoparticles (Mn-SNPs) immune cell recruitment by inducing ROS production which recruits immune cells to the site of administration and induces DC maturation. Manganese ions also allowed for efficacious antigen uptake due to their charge [69].

### 7.3. Photothermal Vaccines

Hyperthermal therapy is a non-invasive cancer treatment modality that has been shown to induce anti-tumor effects by direct cytotoxicity as well as its effect on the tumor microenvironment [65]. In addition, hyperthermia has been found to enhance the effects of chemotherapy and radiation therapy against certain tumors [91]. Recently, reports have also provided preclinical and patient data supporting positive immune modulatory effects of hyperthermia [65]. A study by Liu and colleagues [92] has shown the utility of nanotechnology-based photothermal therapy (PTT) to enhance the activity of a tumor vaccine (hEX@BP) prepared by encapsulating black phosphorus quantum dots (BPQDs) with exosomes (hEX) in the lung cancer model. Another study combined BPQDs with neoantigens in liposomes to produce a more efficacious immune response from a vaccine [15]. The gel containing the liposomes contained GM-CSF which primes T cells for activation while recruiting antigen-presenting cells (DCs). Upon 808 nm light exposure, the black phosphorus heats up and ablates the gel. This vaccine was found to effectively halt tumor growth and, in combination with PD-1 checkpoint inhibitors, proved successful in eliminating tumors [15]. Another study evaluating hyperthermal anti-cancer therapy coated black-phosphorus quantum dot nanoparticles with autologous resected tumor cell membranes and loaded it into a thermosensitive gel containing GM-CSF and LPS which are meant to enhance dendritic cell activity [16]. In combination with PD-1 checkpoint inhibitors, the injection increased tumor-specific CD8+ T cell anti-cancer activity, preventing metastasis in a mouse model [16].

### 7.4. Liposome/Lipoplex

Cationic liposomes can form complexes with DNA or RNA for a novel mechanism of gene transfer that aids the stability of genetic material and increases uptake [17]. Salomon et al. [17] delivered an RNA-lipoplex (RNA-LPX) vaccine that was capable of inducing CD8+ T cell response via delivery of genetic material encoding for CD4+ T cell-recognized neoantigens. Combined with local radiotherapy, the two treatments had further synergistic effects, as opposed to local radiotherapy alone in which most CD8+ T cells targeted the immunodominant gp70 antigen commonly found in the tumor cells [17]. Furthermore, the treatment regimen combined with anti-CTLA4 checkpoint inhibitors enhanced the response with complete remission of gp70-negative mice, with all treated mice surviving. Kranz et al. [93] used an experimental mouse model to demonstrate that RNA-LPXs are effective vehicles to transport RNA to DCs and eliciting a strong anti-cancer response via IFN-α-dependent pathways. The RNA-lipoplexes encoding mutant neoantigens or endogenous self-antigens elicited effector and memory T cell responses. The two studies together demonstrate the diverse effects of neoantigen vaccines delivered via lipoplexes, providing the rationale for further research and development of this approach for effective cancer vaccine strategies in the future.

## 8. Mechanistic Features of Neoantigen Vaccine Effects

A number of recent studies have focused on understanding the key cellular components and mechanisms that may influence the overall efficacy of neoantigen vaccines. One study found that no matured neoantigen-specific T cells were present prior to vaccination despite clonal neoantigens being adequately present [94]. Upon DC vaccination, T cell responses were elicited and composed of several TCR clonotypes. A proof-of-concept study found that mice injected with a DNA vaccine based on autologous tumor neoantigens and subsequently pulsed via electroporation, elicited predominantly MHC-I response, inducing CD8+ T cells [95], providing rationale for future use of DNA neoantigen vaccines.

Despite evidence of the necessity of cytotoxic CD8+ responses for tumor clearance, there is growing evidence that CD4+ T cells are also necessary for clearing and controlling tumor growth. It has been found that CD8+ T cells were effective at clearing tumors with both MHC-I and MHC-II antigens locally; however, distant tumors that lack MHC-II epitopes remain challenging [96], demonstrating that CD4+ T cell responses are vital in neoantigen-related clearance of tumors. Similar findings support the critical involvement of tumor-specific CD4+ T cells in non-small cell lung cancer [97].

Studies are currently in progress to elucidate the mechanisms by which neoantigens are recognized and responded to in vivo. For example, it has been found that neoantigen-reactive CD8+ T cells are also those that are positive for PD-1, not only suggesting the possibility of utilizing these tumor-reactive T cells for future neoantigen identification, but also for elucidating the immunological mechanisms in which cancer cell tolerance still results despite appropriate neoantigen recognition. In addition, studies have also identified a novel class of neoantigens that have no detectable non-synonymous mutations. For example, novel neoantigens from dysfunctional transporter associated with antigen processing (TAP) [59], as well as phosphorylated peptides are demonstrating the potential to be therapeutic targets in certain cancer systems [60]. These advances highlight the areas for key future studies for effective neoantigen-based therapeutics development in oncology.

Previous knowledge of molecular mimicry has recently been translated to neoantigen research and it has been shown that the phenomenon may contribute to a more robust immune response based on both increased progression-free survival and cross-reactivity of immune cells with both HCMV peptide homologues and melanoma cells [55]. Working on colorectal and endometrial cancer models with microsatellite instability (MSI), Ballhausen et al. [37] have found a negative correlation between frameshift mutation frequency and the predicted immunogenicity of the peptides formed, indicating a possible process of counterselection of tumor cell clones having frameshift peptides with high immunogenicity. However, distinct outlier mutations were also noted that are related to immunogenic frameshift peptides, suggesting a driver function during tumor clonal evolution [37]. These data indicated the high capacity of neoantigens formed from shared mutations in the vaccine development for MSI tumors.

### 8.1. Translational Viability

Research efforts have been established to explore the feasibility of neoantigen-based immunotherapeutic approaches in a wide spectrum of cancer types. These include the expansion of research studies to include common high-risk malignancies such as gastric [42], colorectal [40], bladder [98], and lung cancer [99].

Medulloblastoma is a cancer with a low mutational load and would intuitively be a low yield target for neoantigen vaccination. Blaeschke et al. [41], however, found at least two immunogenic neoantigen targets in pediatric medulloblastoma tumor specimens, and determined potential biomarkers associated with CD8+ T cell response. This supports the feasibility of neoantigen vaccination based therapeutic strategies also in cancers with low mutational loads, despite being considered as potentially not easily targetable. However, it has been shown that a low mutational burden in some cancers, such as ovarian cancer, can be challenging to generate effective neoantigen immunotherapies [100]. Conversely, there are, however, several cancers with high mutational loads that have yet to be substantiated for their feasibility for possessing effective of neoantigens as therapeutic targets [101].

Supporting current clinical efforts to bring neoantigen vaccination to hematological cancers [26], Stroopinsky et al. [18] found that an acute myeloid leukemia (AML)-fused DC vaccine overcame tumor resistance to a PD-L1 checkpoint inhibitor therapy in an AML murine model. Not only is this important in adding to current knowledge of vaccine production, the translation of neoantigen vaccine technology to refractory hematological malignancies is critical to address an unmet need in current oncology.

### 8.2. Neoantigen Biomarkers

Current literature uses a variety of metrics to determine the efficacy of neoantigen-targeted therapies, with many clinical studies reporting vaccine-related immune responses compared to objective clinical responses [34,102,103,104,105]. Efforts are being made to address the lack of correlative biomarkers related to neoantigens associated with efficacy and prognosis. One recent study introduced a method of detecting responses that may be a more direct indicator of T cell reactivity than current methods of measuring cytokine profiles or cellular cytotoxic activity [106]. Using NGS, expression of specific T cell receptors for neoantigens was measured, as being directly indicative of neoantigen-T cell reactivity. As opposed to directly measuring T cell response, another study found that a notable correlation between circulating CX3C chemokine receptor 1 (CX3CR1) levels and neoantigen specific T cells exists as demonstrated in murine model models [107].

There have also been attempts to characterize prognosis by correlating neoantigen burden in the clinical context. In a retrospective analysis of patients with hepatocellular carcinoma, a neoantigen predicting algorithm was compared to patient survival [108]. In this particular investigation, somatic mutational load and number and quality of neoantigens had no correlation with increased overall survival in patients; however, in the context of patients with high levels of granzyme A, a correlation was found between neoantigen quality and quantity and survival. Another study found no prognostic correlation of immune status based on neoantigen presence, and, instead, it was found that a generally high level of TIL diversity was more indicative of immune status [72]. In contrast, a number of emerging studies have shown that neoantigen burden is a strong correlate of prognosis [109,110]; however, it has also been shown that neoantigen abundance should not be used alone as a prognostic marker and has been found to be more indicative of survival when combined with infiltrating CD8+ T cell count [111]. It should be noted that the heterogeneity of findings may be due, in part, to the contributing biological variables in the distinct cancers being studied, using, for example, glioblastoma and hepatocellular carcinoma [72,108], versus clear cell renal cell carcinoma, bladder cancer, and pancreatic cancer [109,110,111]. Additional focused research is needed to identify the biological markers and correlates that may determine the strength of the overall response that would result from neoantigen vaccine treatments.

### 8.3. Vaccine Production

Personalized anti-cancer vaccine generation poses unique challenges compared to the formulation of other types of vaccines. The process must address and validate the various distinctive steps in the designing, manufacturing, and administration processes to maximize the effectiveness and safety of the vaccines. For example, the AML-DC hybridoma vaccine represents a traditional method of producing immune activation, however, it demonstrated the capability to overcome tumor tolerance to checkpoint inhibitors [18]. Additionally, Mac Keon et al. [112] investigated the use of syngeneic cells to produce DC vaccines. It was found that using syngeneic cells to make a DC vaccine produced a longer lasting, and more potent, anti-tumor response as opposed to using allogeneic cells [112]. This warrants further investigation in human-derived tumor tissues; however, the evidence from this study supports the current method of producing DC vaccines using autologous DCs. Less traditional methods have also shown promise. For example, Horrevorts et al. [113] utilized extracellular vesicles formed in apoptotic melanoma cells followed by the modification of the outer glycocalyx with high-mannose glycans, which are easily recognized by DC receptors. It was found that the use and modification of the extracellular vesicles with high-mannose glycans improved vaccine uptake when compared to unmodified vesicles [113].

Recently, Zhang et al. [24] compared the efficacy of a personalized neoantigen-adjuvant peptide vaccine with a personalized neoantigen-pulsed dendritic cell vaccine in mice. Not only did the DC vaccine induce increased IFN-γ levels in all six mice (compared to four of six mice in the control vaccine), but the DC vaccine also demonstrated higher CD8+ IFN-γ-positive tumor infiltrating cells in five of six mice (compared to two of six mice for the adjuvant group) [24]. Further studies are needed to investigate the factors that may determine response rates in allogeneic DC vaccines.

## 9. Clinical Evaluation

The clinical efficacy of neoantigen vaccination has been studied most extensively in the context of melanoma [21,103,104,114,115,116]. However, recent clinical studies and trials have reported findings on neoantigen-based vaccine treatments in other solid tumors, such as glioblastoma [102,117,118] and ovarian cancer [119,120,121] (Table 1). Given experimental evidence supporting the usage of neoantigen vaccination, even in cancers with low mutational burdens, the extension of research to varying forms of cancer has been predictable. Presently, some studies have reported indirect evidence for benefit such as increased immune activity measurements, while a number of others have reported measurable clinical responses in patients. In addition, certain cancer-specific findings and symptoms such as bone pain, respiratory discomfort, and CA-125 levels have been alleviated post-vaccination [120,122]. Additionally, responses are reported over a variety of cancers, with one vaccine being utilized in a pan-cancer study and reporting a 71.4% disease control rate of stable tumors [122]. Moreover, clinical studies have begun incorporating the use of neoantigen vaccination either as an adjuvant or with an adjuvant based on rationale provided by experimental evidence [15,16,17,18,19,20,21,77,78,83,94,98].

An mRNA vaccine synthesized for six different patients contained 13–20 predicted neoantigens based on the whole exome sequencing of the patients and administered to patients following resection of their stage 3 melanoma [21]. Four of six treated patients remained disease-free for the remainder of the study, with the other two having untreated lung metastases showed complete radiographic response with the checkpoint inhibitor pembrolizumab. Similarly, in a clinical trial for patients at high risk of relapse of melanoma, patients were administered a personalized mRNA vaccine meant to prevent recurrent disease [104]. Out of 13 patients, eight remained recurrence-free for the remainder of the study period and these patients experienced strong immune responses, demonstrated by vaccine-induced tumor infiltrating T cells being present in two patients’ resected metastases. Two of the five patients who had relapsed experienced vaccine-related responses. The patients exhibited evidence of poly-specific immune response, indicating that their immune system recognized several tumor antigens post-vaccination and suggesting a diverse immune response with possible “neoantigen spreading”. Additionally, the vaccine induced de novo CD4+ and CD8+ T cell responses. Interestingly, it has been found that a DC vaccine generated with synthetic peptides produced an immune response with similar breadth and diversity [114]. There was evidence that T cells with a naïve phenotype exist for specific sub-dominant neoantigens but first needed to be induced to an effector phenotype. The authors also suggested that subdominant neoepitopes should be included in all future neoantigen vaccines to avoid selection of antigens and outgrowth of abnormally expressed neoantigens, addressing possible concerns for counterselection of neoantigens [37].

In a long-term follow up of surgically resected melanoma patients who had received a long-peptide neoantigen vaccine (NeoVax), it was found that six of eight patients had no signs of disease and the study identified the presence of diversified neoantigen-specific T cells in these patients [115]. Additionally, evidence was found of epitope spreading as well as the generation of long-term T cells. The presence of long-term, memory phenotype T cells has been found in various other studies [93,104,115,118,123]. However, the polyfunctionality of vaccine-related T cells is relatively unstudied and warrants further investigation [77]. Another long-term study, conducted as a 5-year follow up to a clinical trial, compared the efficacy of an autologous DC vaccine to the efficacy of autologous irradiated tumor cell vaccine [116]. When compared to an irradiated autologous tumor cell vaccine, the DC vaccine was associated with longer overall survival, with a median of 43.4 months compared to 20.5 months, and a 70% reduction in risk of death.

Another study found evidence for immune activity in a combination of DC vaccine and neoantigen reactive T cell therapy in patients with a variety of refractory solid tumors [30]. Two methods of identifying candidate peptides were used: the first method of neoantigen matching was by identifying mutant peptides with high variant frequency and the peptides with high predicted HLA binding affinity were synthesized; the second was that patients’ neoantigens were matched to a neoantigen peptide library that was made for the study and neoepitopes were identified by matching hotspots in the library, then producing memory T-cell responses in vitro. After vaccination, a patient went into complete remission with evidence of epitope spreading, another patient had a 2.9 month immune-related partial response, and the remaining four patients experienced progression-free survival with a median of 8.6 months. The method of neoantigen identification used in this study resulted in a significantly reduced number of candidate peptides [30], which may result in more efficient and accessible production of neoantigen-targeting therapies in the future.

An individualized dendritic cell-tumor cell hybridoma vaccine for AML echoed the findings of other studies about the extent of immune response observed [26,114,119]. It was found that the vaccine produced wide coverage T cell immunity through proliferation of cancer-recognizing T cells. The treatment resulted in the proliferation of both CD4+ and CD8+ T cells, being suggestive of DC fused cells’ ability to produce a variety of tumor-associated antigens that can be recognized by the immune system. Combined with the knowledge that DC vaccines can overcome checkpoint inhibitor resistance in murine models of AML [18], a target population for future AML DC vaccine clinical trials could be those whose tumors have developed resistance to checkpoint inhibitor therapy.

Many recent clinical trials for neoantigen vaccination have investigated its application to glioblastoma, particularly focusing on how a low mutational burden and an immunologically “cold” microenvironment may influence the effectiveness of this approach [41]. Similarly, a trial was conducted with a peptide-based vaccine called GAPVAC-101 in patients with newly diagnosed glioblastoma combined with regular chemoradiotherapy following resection [102]. The trial had two different treatments, APVAC1, which was directed towards unmutated antigens, and APVAC2, which targeted neoepitopes specific to patients. Analysis of the trial data found that reactivity to neoepitope-specific T cells was induced for at least one neoepitope in 8 out of 10 patients. It was also found that reactive T cells were present prior to vaccination in low counts and in naïve phenotypes, representing a finding consistent across other recent studies [97,114,119]. In a large phase III clinical trial for DCVax-L (Northwest Biotherapeutics), an autologous tumor lysate dendritic cell vaccine in patients with newly diagnosed glioblastoma, the vaccine was found to be safe and showed efficacy in extending overall survival of treated patients [117]. The vaccine was used in patients with newly diagnosed glioblastoma who had received standard treatments-resection, radiotherapy, and temozolomide-hinting at the adjuvant capability of certain neoepitope vaccines. Keskin et al. [118] found that a supplementary personalized vaccine generated a robust immune response in patients following resection and radiotherapy. For two patients who did not require dexamethasone, the immune response demonstrated memory and effector phenotypes in CD8+ T cells and higher amounts of TILs. However, patients who had received dexamethasone had no response to the vaccination. It was also noticed that the patients who did not require dexamethasone had reduced expression of regulatory T cells when compared to those who received dexamethasone. The authors noted that significant CD4+ T cell recognition of neoantigens was achieved in the two patients, despite using MHC-I prediction algorithms as opposed to MHC-II. This highlights the significant gap in research data addressing the exact mechanisms of action of neoantigen vaccines.

The vaccine Neo-DCVac (Sichuan University) was tested in 12 patients with advanced lung cancer and was found to have tumor-limiting effects [124]. The objective effectiveness rate, measured as the rate of tumors that had a negative percentage change in size below −30%, was 25%. The disease control rate, however, was 75%, and was measured as the rate of tumors that had a percentage change in size less than 20%. Although the study used a small sample size, the obtained rates are encouraging given all participants had heavily treated metastatic lung cancer. It was noted that, of the six patients whose PBMCs responded to eight or more neoantigens, one patient achieved an objective response and five others achieved disease control. This may indicate a prognostic association with T cell reactive neoantigens, agreeing with other literature [109,110].

In agreement with several other studies, the Oxidized Cell Dendritic Cell vaccine (OCDC) was found to elicit an efficient immune response in ovarian cancer [119]. Additionally, patients treated with cyclophosphamide experienced greater immune responses and longer overall survival times similar to findings reported previously with the use of cancer vaccines as adjuvant therapies. The authors also suggested that the vaccine activated naïve T cells with existing unknown neoantigen receptors, due to the presence of neoantigen-reactive T cells with an immature phenotype. Another trial done on ovarian cancer studied a patient with recurrent and refractory ovarian cancer who was administered a neoantigen peptide-based dendritic cell vaccine [120]. Accordingly, the patient’s CA-125 levels started to decline, and tumor cells found in ascites declined as well. The authors also noted the prompt resolution of the tumor associated symptoms. Further research studies are planned in patients with ovarian cancer given the proposal for a phase I/II trial involving patients with advanced ovarian cancer [121]. The trial aims to compare the efficacy of an autologous DC vaccine generated by pulsing the DCs with peptides and an autologous DC vaccine generated by growth in autologous tumor lysate.

However, a clinical trial for a tumor-RNA whole cell DC vaccine for advanced renal cell carcinoma with concurrent sunitinib treatment was ended early due to lack of efficacy in 2017 [125]. Nevertheless, immune responses were detected in 70% of patients with the magnitude of immune response being correlated to overall survival [126]. While the trial was ended early, the results are consistent with literature supporting evidence of vaccine-related immune responses above normal levels [34,101,102,103,105], and statistical analysis found a correlation between the magnitude of immune response and overall survival [126].

## 10. Current Limitations of Neoantigen-Based Vaccines

### 10.1. Clonal Evolution and Immune Evasion

A large proportion of neoantigens are encoded by mutations that occur in passenger-type genes, and such mutations may occur only in certain tumor clones as ‘subclonal’ epitopes. Neoepitopes that arise from passenger mutations may elicit immunological responses against the tumor, but such mutations can be lost during clonal evolution as their presence does not provide a selective survival advantage for the cancer. It is ideal to target mutations of clonal status to fully recognize and eradicate tumor cells, however, the high degree of spatial and genetic diversity of tumors, especially in advanced cancers, makes it difficult to ascertain that a mutation is in fact clonal, and the methods used to identify clonal mutations remains to be challenging and resource intensive on a per-patient level.

In addition to the potential loss of neoantigens, tumor cells may also possess immune escape mechanisms that can reduce the efficacy of cancer vaccines. These mechanisms include defective antigen presentation, suppression of immune checkpoints via upregulation of inhibitory immunoreceptors (such as PD-1 and CTLA-4), and the presence of immunosuppressive mediators within the tumor microenvironment. As we have mentioned, the use of immune checkpoint inhibitors is being explored as a concurrent therapy with neoantigen-based vaccines in clinical settings, which may effectively allow for tumor-specific immune responses to overcome tumor-associated immunosuppression.

### 10.2. Cost Optimization

The current high costs and complexity associated with the generation and delivery of neoantigen-based vaccines are also barriers for the widespread adoption of this therapeutic approach. These aspects highlight the need for continued development through collaborative efforts between public and private institutions to identify cost-effective systems that can streamline the processes associated with neoantigen identification, vaccine manufacturing, and delivery, to ensure the timely deployment of these therapies for the most optimal benefit to patients. By optimizing current workflows and pursuing innovative platforms, such as automated and accurate prediction models for neoantigen identification, novel delivery mechanisms, and the development of rapid and efficient synthesis methods, the cost- and time-to-production of neoantigen-based vaccines should be dramatically improved.

## 11. Conclusions

Neoantigen vaccination maintains its promise as a potentially effective anticancer treatment modality, as it did through the start of the 21st century, augmented by increasing clinical evidence of its adjuvant capabilities. Although clear clinical responses were not demonstrated in all completed clinical trials, there is significant evidence to indicate the potential of neoantigen-based vaccines to enhance the outcomes in a number of difficult to cure malignancies. Studies that are in progress, particularly with respect to identifying novel bioinformatics and biosynthetic pathways to generate highly effective vaccine preparations, in addition to helping to understand its role as an immune adjuvant, would support the formulation of effective clinical trials in the future.

## Figures and Tables

**Table 1:** Summary of clinical evaluations related to neoantigen vaccination.

Cancer Type	ClinicalTrial.gov Identifier	Phase	Formulation	Route	Additional Intervention	Outcome
Melanoma	NCT01970358	I	Poly-ICLC (NeoVax)	s.c.	Pembrolizumab ^a^	6/8 (75%) NED
	NCT02035956	I	Polyepitope coding RNA (IVAC MUTANOME)	i.n.	Pembrolizumab ^a^	8/13 (62%) NED
	NCT00683670	I	DCV	i.v.	Cyclophosphamide	Ex vivo results published [114]
	NCT00948480	II	DCV	s.c.	NA	43.4 mth m. OS 70% reduced RR (vs. TCV group)
			TCV	s.c.	NA	20.5 mth m. OS
Glioblastoma	NCT02149225	I	Poly-ICLC (GAPVAC)	i.d.	Temozolomide	14.2 mth m. PFS 29 mth m. OS
	NCT02287428	I	Poly-ICLC	s.c.	Radiotherapy	7.6 mth m. PFS 16.8 mth m. OS
	NCT00045968	III	DCV (DCVax-L)	i.d.	Temozolomide	23.1 mth m. OS
Ovarian cancer	NCT01132014	I	DCV (OCDC)	i.n.	Bevacizumab Cyclophasphamide	13/25 (52%) SD 14 mth m. PFS
	NCT02933073	I	SLP (OncoImmunome)	NA	Chemotherapy	Recruitment on-hold
Advanced lung cancer	NCT02956551	I	DCV (Neo-DCVac)	s.c.	Cyclophosphamide	5.5 mth m. PFS 7.9 mth m. OS
Metastatic renal cell carcinoma	NCT01582672	III	DCV (Rocapuldencel-T)	i.d.	Sunitinib ^b^	6 mth m. PFS (vs. 7.83 mth m. PFS SOC group) 27.7 mth m. OS (vs. 32.4 mth m. OS SOC group) Terminated due to lack of efficacy
Solid tumors	NCT03662815	I	SLP (iNeo-Vac-P01)	s.c.	NA	4.6 mth m. PFS 12-mth OS 55.1%
	NCT02721043	I	Poly-ICLC (PGV-001)	i.m.	Lenalidomide	4/12 (33%) NED 20.3 mth m. PFS
Multiple cancers	NCT02897765	I	Poly-ICLC (NEO-PV-01)	s.c.	Nivolumab	23.5/8.5/5.8 mth m. PFS (MEL/NSCLC/BC) 20.7 mth m. OS (BC)

^a^ Administered to patients with disease reoccurrence. ^b^ Administered to patients in combination with Rocapuldencel-T, or alone as the SOC. Abbreviations: BC, bladder cancer; DCV, dendritic cell vaccine; i.d., intradermal; i.m., intramuscular; i.n., intranodal; i.v., intravenous; m., median; MEL, melanoma; NA, not applicable; NED, no evidence of disease; NSCLC, non-small cell lung cancer; OS, overall survival; PFS, progression free survival; s.c., subcutaneous; TCV, tumor cell vaccines; RR, relative risk of death; SD, stable disease; SLP, synthetic long peptides; SOC, standard of care.
